# Effects of cold water immersion versus active recovery on oxidative stress, redox gene expression and mitochondrial adaptations after strength training

**DOI:** 10.1113/EP093660

**Published:** 2026-07-22

**Authors:** Llion A. Roberts, Chantal A. Pileggi, Nina Zeng, Vandre C. Figueiredo, James F. Markworth, Truls Raastad, David Briskey, Katsuhiko Suzuki, Jeff S. Coombes, David Cameron‐Smith, Jonathan M. Peake

**Affiliations:** ^1^ School of Human Movement and Nutrition Sciences The University of Queensland Brisbane Queensland Australia; ^2^ Australian Centre for Precision Health and Technology (PRECISE) School of Allied Health, Sport and Social Work Griffith University Gold Coast Queensland Australia; ^3^ Liggins Institute The University of Auckland Auckland New Zealand; ^4^ Department of Biochemistry, Microbiology and Immunology University of Ottawa Ottawa Ontario Canada; ^5^ Department of Biological Sciences Oakland University Rochester Michigan USA; ^6^ Department of Animal Science Purdue University West Lafayette Indiana USA; ^7^ Norwegian School of Sport Sciences Oslo Norway; ^8^ RDC Global Brisbane Queensland Australia; ^9^ Faculty of Sport Sciences Waseda University Tokorozawa Japan; ^10^ Riddet Institute Massey University Palmerston North New Zealand; ^11^ School of Biomedical Sciences Queensland University of Technology Brisbane Queensland Australia

**Keywords:** adaptation, exercise, recovery

## Abstract

We compared the acute and chronic effects of postexercise cold water immersion (CWI) and active recovery (ACT) on oxidative stress, redox‐related gene expression, mitochondrial adaptations and muscle oxidative capacity. In a crossover study, nine males completed strength sessions, followed by either 10 min CWI or ACT. There were time effects for blood oxidative stress markers, including serum derivatives of reactive oxygen metabolites and serum biological antioxidant potential (*P *< 0.05). F_2_‐Isoprostanes were lower after exercise in the CWI trial compared with the ACT trial (*P *< 0.05), whereas plasma glutathione peroxidase activity was higher in the CWI trial at 2 h after exercise (*P *< 0.05). There were time effects for redox‐related genes, including *NRF2*, *SXRN*, *HMOX1*, *GCLM*, *TP53* and *TXN*, in muscle after exercise (*P *< 0.05). Postexercise gene expression did not differ between the CWI and ACT trials. In a parallel‐group study, 21 males strength‐trained twice a week for 3 months and performed either 10 min CWI or ACT after each session. There were time effects for the activity of citrate synthase and mitochondrial enzyme complexes I and IV (*P *< 0.05) and the protein abundance of p‐AMPK (*P *< 0.05) in muscle after training. These responses did not differ between the CWI and ACT groups. Evaluation of muscle oxidative capacity using near‐infrared spectroscopy showed slower oxygen desaturation during a 10 s isometric contraction, and greater oxygen consumption during 50 isokinetic contractions after training in the CWI group (*P *< 0.05). In conclusion, CWI after resistance exercise does not alter acute redox‐related transcription or chronic mitochondrial adaptations, whereas it might improve muscle oxygen kinetics.

## Abstract (highlights)

- **What is the central question of this study?**
  Cold water immersion is widely used after exercise, but whether it alters redox signalling and mitochondrial adaptations to resistance training is unknown.
- **What is the main finding and its importance?**
  By combining an acute resistance exercise study with a 3 month training intervention, we show that cold water immersion does not attenuate exercise‐induced oxidative stress responses, redox‐related gene expression or mitochondrial enzyme adaptations compared with active recovery. Instead, cold water immersion improves muscle oxygen delivery and utilisation during exercise, indicating enhanced oxidative capacity. These findings show that postexercise cooling does not impair key adaptive pathways and might, in some contexts, support muscle metabolic function.

## 1 INTRODUCTION

Redox signalling during exercise is an important factor affecting skeletal muscle adaptations to exercise training (Margaritelis et al., 2018; Merry & Ristow, 2016). Reactive oxygen species, such as the superoxide anion (·O_2_^−^) are formed in skeletal muscle by electron chain complexes I and III (C‐I and C‐III), and by the activity of NADPH oxidase and xanthine oxidase (Egan & Zierath, 2013). Reactive oxygen species can stimulate mitochondrial biogenesis in skeletal muscle directly by phosphorylating p38 mitogen‐activated protein kinase and indirectly by phosphorylating adenosine monophosphate‐activated protein kinase (AMPK) (Hood et al., 2016). As a proxy for reactive oxygen species production, markers of blood oxidative stress (e.g., thiobarbituric acid reactive substances, lipid hydroperoxides, malondialdehyde, 8‐hydroxy‐2‐deoxyguanosine, advanced oxidation protein products, protein carbonyls, glutathione, glutathione peroxidase, catalase and total antioxidant capacity) increase after resistance exercise (Deminice et al., 2010; Goldfarb et al., 2005, 2008; Hudson et al., 2008; Mohammadjafari et al., 2019; Rietjens et al., 2007). Antioxidant enzyme activity also increases in skeletal muscle after exercise (Rietjens et al., 2007), reflecting part of the broader redox response to contractile activity. Together, these oxidative and antioxidant changes contribute to redox signalling and might stimulate mitochondrial protein synthesis (Donges et al., 2012; Wilkinson et al., 2008), mitochondrial enzyme activity (Tang et al., 2006) and respiration (Porter et al., 2015; Salvadego et al., 2013) in skeletal muscle after resistance training. These redox‐sensitive pathways provide a mechanistic basis for how the oxidative environment during exercise can influence longer‐term mitochondrial and metabolic adaptations in skeletal muscle.

Most research has focused on mitochondrial biogenesis following aerobic training (Perry & Hawley, 2018), but resistance exercise also induces mitochondrial protein synthesis in skeletal muscle (Donges et al., 2012; Wilkinson et al., 2008). Evidence supporting long‐term changes in mitochondrial biogenesis after a period of resistance training is equivocal (Groennebaek et al., 2018; Haun et al., 2019; Irving et al., 2015; Kon et al., 2014; Lamb et al., 2020; Lim et al., 2019; Mesquita et al., 2020; Pesta et al., 2011; Porter et al., 2015; Robinson et al., 2017; Salvadego et al., 2013; Tang et al., 2006; Wilkinson et al., 2008). This variation is attributable, in part, to differences in the methods/assays used to assess mitochondrial content and function (e.g., enzyme activity, mitochondrial protein abundance, mitochondrial respiration or mitochondrial volume) (Parry et al., 2020) and the small sample sizes in these studies (Groennebaek & Vissing, 2017). Some of the inconsistencies in mitochondrial biogenesis markers might also reflect individual variability in redox responses during and after resistance exercise.

The role of reactive oxygen species as molecular messengers that regulate many physiological processes, including mitochondrial biogenesis, has been contrasted widely with their purported negative effects on muscle function and tissue damage. Related debate has focused on whether strategies that counteract exercise‐induced oxidative stress might inadvertently disrupt redox‐sensitive signalling (Merry & Ristow, 2016). These issues are relevant here because postexercise recovery treatments might alter the oxidative environment in which early signalling processes occur. Research on how recovery treatments, such as cold water immersion (CWI), influence oxidative stress and muscle redox signalling is limited (Coelho et al., 2021; de Freitas et al., 2019; Leeder et al., 2019). Mechanistically, performing CWI after exercise could, in theory, influence systemic and local oxidative stress by reducing venous blood O_2_ saturation (Roberts et al., 2014), decreasing muscle blood flow (Mawhinney et al., 2017) and altering muscle perfusion and metabolic activity (Ihsan et al., 2013; Roberts, Muthalib et al., 2015). Because redox‐related signals help to initiate mitochondrial biogenesis, any alteration of these early responses by regular recovery treatments could also influence longer‐term adaptations.

Evidence from animal studies shows that CWI (Puntel et al., 2011, 2013; Silva et al., 2016) and icing (Siqueira et al., 2017) can attenuate oxidative stress in injured skeletal muscle. In humans, exposure to cold air (Slivka et al., 2013) or cold water (Allan et al., 2017; Ihsan et al., 2014; Joo et al., 2016) after endurance exercise can enhance the expression of proliferator‐activated receptor gamma coactivator (PGC)‐1α mRNA and the abundance of AMPK^Thr172^ protein phosphorylation in skeletal muscle. In contrast, cold application by topical icing does not alter the expression of other genes involved in mitochondrial biogenesis (e.g., mitochondrial transcription factor A, nuclear respiratory factor 1 and 2, and oestrogen‐related receptor α) (Shute et al., 2017). It is currently unknown how CWI after resistance exercise influences acute redox‐regulated factors in skeletal muscle or how any such effects influence chronic mitochondrial adaptations after resistance training.

To address these gaps, we compared the effects of CWI and ACT on: (1) short‐term changes in blood oxidative stress markers and redox‐related gene expression in skeletal muscle following a single session of resistance exercise; and (2) long‐term adaptations in mitochondrial enzyme activity, regulators of mitochondrial biogenesis, and muscle oxidative capacity following 3 months of resistance training. By examining acute redox‐related responses and chronic adaptations within the same experimental framework, this study provides an integrated perspective on whether early oxidative signalling might influence longer‐term changes in mitochondrial function and muscle oxygenation. We hypothesised that, compared with ACT, CWI would attenuate acute increases in oxidative stress and redox‐related gene expression and would blunt long‐term improvements in mitochondrial enzyme activity, mitochondrial biogenesis signalling and muscle oxidative capacity.

## 2 MATERIALS AND METHODS

### 2.1 Ethical approval

The study participants were informed about the requirements and possible risks of the study before providing their written informed consent. The study conformed to the standards set by the *Declaration of Helsinki*, except for registration in a database. The experimental procedures for the study were approved by the Human Research Ethics Committee at the University of Queensland (#2013000306).

### 2.2 Study design

**FIGURE 1:**
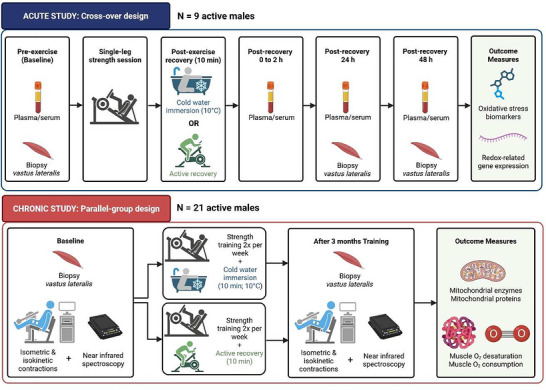
Schematic diagram of protocols for acute and training studies. See the Materials and Methods section for more details. This figure was created using Biorender.com.

The study reported here is part of a larger project, details of which have been published elsewhere (Roberts, Raastad et al., 2015), with additional measurements included specifically for this study to support muscle oxygenation and oxidative capacity assessments. In brief, the study consisted of two parts that involved an acute resistance exercise session and 3 months of resistance training (see Figure 1). The blood and muscle samples used for analysis in the present investigation were collected from the same participants in the larger project.

The acute exercise study involved a randomised, cross‐over study in which nine healthy and physically active young males (mean ± SD: age, 22.1 ± 2.2 years; height, 1.80 ± 0.06 m; and body mass, 83.9 ± 15.9 kg) completed two sessions of resistance exercise ≥1 week apart, followed by either CWI or ACT. Blood and muscle tissue were sampled before and after exercise in each session.

The training study involved a randomised, controlled trial, in which 21 healthy and physically active young males were matched for strength and lean body mass, then randomly assigned to a CWI group (*n* = 11; mean ± SD: age, 21.2 ± 2.2 years; height, 1.81 ± 0.1 m; and body mass, 81.3 ± 11.6 kg) or an ACT group (*n* = 10; mean ± SD: age, 21.3 ± 1.9 years; height, 1.83 ± 0.1 m; and body mass, 79.2 ± 4.4 kg). All the participants performed lower‐body strength training twice a week for 3 months and completed their respective recovery treatment after each training session. Muscle was sampled at rest before the first training session and after the final training session. Near‐infrared spectroscopy (NIRS) measurements of muscle perfusion and oxidative capacity were performed on the vastus lateralis muscle of one leg, at rest, 10−14 days before the first training session and 48 h after the final training session.

### 2.3 Acute resistance exercise protocol

The resistance for each exercise in the acute study was established during an initial session performed 4–5 days before the first trial. During this visit, participants lifted progressively heavier loads to determine their 8–12 repetition maximum (RM) for single‐leg leg press and knee extension, and 12 RM for single‐leg squats and lunges. Participants were then randomly assigned to begin with either CWI or ACT following the first trial, and they completed the alternative treatment after the second trial (see Figure 1). During each trial, participants performed six sets of 8–12 repetitions of single‐leg leg press and knee extension at 8–12 RM, and three sets of 12 repetitions of single‐leg squats and lunges at 12 RM. The exercised leg was alternated between trials.

### 2.4 Three‐month resistance training programme

The participants performed lower‐body strength training two times per week for 3 months and completed their respective recovery treatment after each training session. The resistance training was progressive and involved a combination of leg press, knee extension, knee flexion, walking lunges and plyometrics. The leg press involved six sets each of 8–12 repetitions at a workload of 8−12 RM. The knee extension and knee flexion involved three sets of 12 repetitions at a workload of 12 RM. The walking lunges involved three sets of 10−18 repetitions while carrying dumbbells. Loads corresponding to 8, 10 and 12 RM (leg press) and 12 RM (knee extension and flexion) were assessed at the beginning of session 1, and after every fourth session. In weeks 1–3, the dumbbells weighed the equivalent of 20% body mass, with loading increased progressively every 3 weeks up to an additional 15 kg in weeks 10−12. The plyometrics involved countermovement box and drop jumps, slow eccentric squat jumps and split lunge jumps. These jumps progressed from body mass only in weeks 1–3 to carrying dumbbells weighing 7.5 kg in weeks 10−12.

### 2.5 Recovery treatments

CWI involved seated immersion to the waist in 10°C water for 10 min. Active recovery (ACT) consisted of 10 min of low‐intensity cycling. Treatments commenced within 5 min of exercise.

### 2.6 Blood collection and muscle biopsies

In the acute study, venous blood samples were collected before exercise (PRE), within 5 min after exercise (POST), immediately after each recovery intervention (REC) and at 1, 2, 24 and 48 h after exercise. Blood was obtained from an antecubital vein into lithium heparin and serum separation tubes. Plasma and serum were isolated by centrifugation and stored at −80°C until analysis. Muscle biopsies were obtained from the mid‐portion of the vastus lateralis under local anaesthesia (xylocaine, 10 mg/mL) using a 6‐mm Bergström needle with manual suction. Samples were collected before exercise and at 2, 24 and 48 h after each trial. Biopsies were taken from separate incisions with ∼3 cm spacing to minimise local effects of repeated sampling. Tissue was cleaned of visible fat and connective tissue, rapidly frozen in liquid nitrogen, and stored at −80°C. In the training study, muscle biopsies were obtained using the same procedures from the dominant leg at baseline (4–5 days before training) and 6–7 days after the final training session, with sampling sites separated by ∼3 cm. To avoid interference with NIRS measurements, all biopsies were collected on a separate day and at a site >20 cm proximal to the NIRS measurement location.

### 2.7 Blood analysis

Serum biological antioxidant potential, the concentration of serum derivatives of reactive oxygen metabolites (d‐ROMs) and the concentration of plasma F_2_‐isoprostanes were measured at all eight time points. Plasma glutathione peroxidase activity was measured at five time points (pre‐exercise, immediately postexercise and 2, 24 and 48 h postexercise). Serum d‐ROMs and biological antioxidant potential were measured using established photometric assays (Cornelli et al., 2001; Verde et al., 2002). Plasma F_2_‐isoprostanes concentration was analysed by gas chromatography–tandem mass spectrometry as previously described (Briskey et al., 2014). Plasma glutathione peroxidase activity was measured using a standard coupled enzymatic assay (Wheeler et al., 1990).

### 2.8 Muscle analysis

#### 2.8.1 RT‐PCR

Total RNA was extracted from ∼20 mg of muscle tissue using the AllPrep^®^ DNA/RNA/miRNA Universal Kit (QIAGEN GmbH, Hilden, Germany). Complementary DNA was prepared using a High‐Capacity RNA‐to‐cDNA™ kit (Life Technologies, Carlsbad, CA, USA). Messenger RNA expression was quantified by RT‐PCR on a LightCycler 480 II (Roche Applied Science, Penzberg, Germany) using SYBR Green I Master Mix (Roche Applied Science). Table 1 describes the sequences for the primers that were used in this study. The geometric mean of the housekeeping genes chromosome 1 open reading frame 43 (*C1orf43*), charged multivesicular body protein 2A (*CHMP2A*) and endoplasmic reticulum membrane protein complex subunit 7 (*EMC7*) was used to normalise the data (Vandesompele et al., 2002). Primer pairs were customised using Primer‐BLAST, in which they were designed to avoid intra‐primer homology and secondary structures. Primers were considered optimal if they were 18–24 bases long, had a melting temperature between 55°C and 65°C, and contained a GC content between 40% and 60% (Ye et al., 2012). Standard and melting curves were prepared for each target to evaluate primer efficiency and single product amplification.

**TABLE 1:** Primer sequences for RT‐PCR.

Gene	Forward	Reverse
*NQO1*	AGGACCCTTCCGGAGTAAGA	TGGAAGCCACAGAAATGCAGA
*GSTA*	CGGATGGAGTCCGTGAGATG	GGTGGTTACCATCCTGCAAC
*GSR*	CAGCCCTGGGTTCTAAGACA	CCTTGACCTGGGAGAACTTCAG
*TXN*	CCTTGAAGTAGATGTGGATGACTG	CACCCACCTTTTGTCCCTTCTTA
*TXNRD*	TCATGTGAGGACGGTCGG	GCTTTTGAAGTCTGCCCTCC
*HMOX1*	AAGACTGCGTTCCTGCTCAA	GGGGGCAGAATCTTGCACTT
*SOD1*	GGAAGCATTAAAGGACTGACTGAA	TCCAACATGCCTCTCTTCATCCT
*SOD2*	GTGAACAACCTGAACGTCACC	AGCAACTCCCCTTTGGGTTCTC
*GCLM*	CCCAGATTTGGTCAGGGAGT	GCACTTCTAGTTGATGATGAAGAGT
*GPX*	CCGGGACTACACCCAGATGA	CTTGGCGTTCTCCTGATGCC
*NRF2*	GGTTGCCCACATTCCCAAATC	CGTAGCCGAAGAAACCTCA
*KEAP1*	ACAACAGTGTGGAGAGGTATGAG	TCCTTCGTGTCAGCATTGGG
*SRXN*	GGGCCATGCAGAAGGGATAG	GGCCTTGTAGCTGGTGAGAG
*TP53*	CTAAGCGAGCACTGCCCAA	AAGGCCTCATTCAGCTCTCG

Abbreviations: GCLM, glutamate–cysteine ligase modifier subunit; GPX, glutathione peroxidase; GSR, glutathione reductase; GSTA, glutathione *S*‐transferase alpha; HMOX1, heme oxygenase 1; KEAP1, Kelch‐like ECH‐associated protein 1; NQO1, NAD(P)H quinone dehydrogenase 1; NRF2, nuclear factor erythroid 2‐related factor 2; SOD, superoxide dismutase; SRXN, sulfiredoxin 1; TXN, thioredoxin; TXNRD, thioredoxin reductase.

#### 2.8.2 Mitochondrial analysis

The procedures for homogenising muscle tissue and SDS‐PAGE have been published elsewhere (Roberts, Raastad et al., 2015). After SDS‐PAGE separation in reducing conditions, proteins were transferred to PVDF membranes by semidry transfer (Trans‐Blot Turbo™, Bio‐Rad, Hercules, CA, USA). After 1 h of blocking in 5% bovine serum albumin in Tris‐buffered saline with 0.1% Tween 20 (TBST) at room temperature, membranes were incubated overnight at 4°C with primary antibodies (diluted 1:1000 in TBST) against the following proteins: phosphorylated (p) adenosine monophosphate‐activated protein kinase (p‐AMPK; #2535; Cell Signaling, Beverly, MA, USA); total (t)‐AMPK (#2603; Cell Signaling); PGC‐1α (#Ab3242; Millipore, Cambridge, MA, USA); peroxisome proliferator‐activated receptor α (PPARα; #Ab24509; Abcam, Cambridge, MA, USA); mitochondrial complexes C‐I (NADH:ubiquinone oxidoreductase), C‐II (succinate dehydrogenase), C‐III (cytochrome *bc*_1_), C‐IV (cytochrome *c* oxidase) and C‐V (ATP synthase) (#ab110411; Abcam) and citrate synthase (#Ab96600; Abcam). Membranes were subsequently washed four times for 5 min each in TBST, before they were incubated at room temperature for 1 h with anti‐rabbit or anti‐mouse secondary antibodies (1:5000) linked to horseradish peroxidase (Jackson ImmunoResearch Laboratories, West Grove, PA, USA). Membranes were then washed four times for 5 min each in TBST, before they were exposed on an imaging device (ChemiDoc, Bio‐Rad, Hercules, CA, USA) with enhanced chemiluminescence (ECL Select; GE Healthcare, Little Chalfont, UK). Bands were quantified with Image Lab software (Bio‐Rad). Protein abundance was normalised to GAPDH to account for variation in sample loading and transfer.

#### 2.8.3 Enzymatic analysis

The activity of mitochondrial citrate synthase and mitochondrial complexes C‐I, C‐II, C‐III, C‐IV, C‐V, C‐I+III and C−II+III was measured using a method adapted from the study by Spinazzi et al. (2012) to suit 96‐well microplates. Because the quantity of muscle tissue was limited for some participants, this enzymatic analysis was performed on only eight participants in each group, from whom there was sufficient tissue available to conduct the analysis.

Initially, ∼25 mg of tissue was weighed and homogenised in ice‐cold homogenisation buffer (25 mM TRIS–HCl pH 7.8, 1 mM EDTA, 2 mM MgCl_2_, 50 mM KCl and 0.5% Triton X‐100) using a Qiagen TissueLyser II. Homogenates were then centrifuged at 14 000*g* for 10 min at 4°C. A portion of the supernatant was assayed for total protein content using the bicinchoninic acid assay (Pierce BCA Protein Assay Kit; Thermo Scientific; #23225), and the remaining supernatant was frozen at −80°C until analysis. The activity of individual and paired complexes was normalised to the protein concentrations of each sample.

Citrate synthase activity was determined by measuring the absorption at 412 nm. The citrate synthase assay contained 50 mM Tris–HCl (pH 8.0), 0.2 mM Ellman's reagent (DTNB), 0.1 mM acetyl‐CoA and 0.25 mM oxaloacetate. The rate of change in absorbance and path length in each well of the microplate was determined on a Molecular Devices Spectramax‐340 96‐well micro‐plate spectrophotometer using SoftMax Pro v.3.1.1 (Molecular Devices, CA, USA), with temperature maintained at 25°C. Citrate synthase activity was calculated using an extinction coefficient of 13.6 mmol/cm.

The activity of the mitochondrial complexes was assayed as follows. For C‐I, the assay contained 50 mM K_3_PO_4_ buffer (pH 7.5) with 100 µM NADH, 60 µM ubiquinone_1_, 3 mg/mL fatty acid free‐bovine serum albumin and 300 µM KCN. The decrease in NADH absorbance was measured at 340 nm. For C‐II, the assay contained 25 mM K_3_PO_4_ buffer (pH 7.5), 20 mM succinate, 80 µM dichlorophenolindophenol (DCPIP), 50 µM decylubiquinone, 1 mg/mL fatty acid free‐bovine serum albumin and 300 µM KCN. The reduction of DCPIP was measured by the decrease in absorbance at 600 nm. For C‐III, the assay contained 0.025 % Tween‐20 in 25 mM K_3_PO_4_ buffer (pH 7.5), 100 µM decylubiquinol, 75 µM cytochrome *c*, 500 µM KCN and 100 µM EDTA. The reduction of cytochrome *c* was measured by the increase in absorbance at 550 nm. For C‐IV, the assay contained 50 mM K_3_PO_4_ buffer (pH 7.0) and 50 µM of reduced cytochrome *c*. The oxidation of cytochrome *c* was measured by the decrease in absorbance at 550 nm.

C‐I+III and C‐II+III activities reflect coupled electron transfer through the respective complexes. For C‐I+III, the assay contained 50 mM K_3_PO_4_ buffer (pH 7.5), 200 µM NADH, 50 µM cytochrome *c*, 1 mg/mL fatty acid free‐bovine serum albumin and 300 µM KCN. The reduction of cytochrome *c* was measured by the increase in absorbance at 550 nm. For C‐II+III, the assay contained 0.5 M potassium phosphate buffer (pH 7.5), 10 mM succinate, 50 µM cytochrome *c* and 300 µM KCN. The reduction of cytochrome *c* was measured by the increase in absorbance at 550 nm. The specificity of C‐I to C‐IV assays was verified using established inhibitors: rotenone (10 µM) for C‐I, malonate (10 mM) for C‐II, antimycin A (10 µg/mL) for C‐III, and KCN (300 µM) for C‐IV. These assays were performed on the same spectrophotometer as for citrate synthase, but at 37°C. Activities were calculated using extinction coefficients (ε, in mmol/cm: C‐I, ε = 6.2; C‐II, ε = 19.1; C‐III, *ε* = 18.5, C‐IV, ε = 18.5; C‐I+III, ε = 18.5; and C‐II+III, ε = 13.6).

### 2.9 NIRS assessment of muscle oxygenation and oxidative capacity

Muscle oxygenation was assessed before and after the training intervention using NIRS (PortaMon, Artinis Medical Systems, the Netherlands) positioned over the mid‐belly of the vastus lateralis (∼12 cm above the patella). Probe placement was standardised using anatomical landmarks and replicated between visits. Adipose tissue thickness was accounted for to estimate signal penetration depth, ensuring that the signal reflected intramuscular haemodynamic and metabolic changes.

Participants performed a 10‐s maximal isometric contraction at 70° knee flexion, followed by 50 maximal isokinetic knee extensions (90°/s) on a dynamometer. These protocols were used to assess muscle oxygen desaturation kinetics and oxygen consumption during and after exercise.

NIRS‐derived variables included the rate of muscle oxygen desaturation ($S_{mO_{2}}$ desaturation rate), minimum oxygenation ($S_{mO_{2}}$/min) and mean oxygenation during contractions. These variables provide indices of muscle oxygen utilisation and the balance between oxygen supply and demand.

Muscle oxidative capacity was assessed from the recovery kinetics of muscle oxygen consumption following exercise, using repeated brief arterial occlusions (20 × 10‐s occlusions interspersed with 10‐s reperfusion) applied via an occlusion cuff placed proximally on the thigh. Muscle oxygen consumption was estimated from the rate of deoxygenation during occlusion periods, as previously described (Buchheit et al., 2011; van Beekvelt et al., 2002).

All NIRS data were normalised to a physiological range determined from maximal deoxygenation during arterial occlusion and peak reoxygenation during reactive hyperaemia. Data were sampled at 10 Hz and analysed using software supplied by the manufacturer (Artinis Medical Systems).

### 2.10 Statistical analysis

Statistical analysis was conducted using IBM SPSS Statistics for Windows (v.30.0; IBM Corp., Armonk, NY, USA) and GraphPad Prism v.10.6.1 (GraphPad Software, San Diego, CA, USA). Data were initially checked for normality using the Shapiro–Wilk test. Data that were normally distributed were analysed using repeated‐measures ANOVA. When time or time × trial/group interaction effects were evident, Student's paired *t*‐tests were used to determine changes over time or differences between trials. These data are presented as the mean ± SD. Data that were not normally distributed were initially transformed using the natural logarithm. If the log‐transformed data were normally distributed, they were analysed as described above. These data included: sulfiredoxin and heme oxygenase 1 mRNA expression; mitochondrial enzyme complex C‐II activity; and peroxisome proliferator‐activated receptor gamma coactivator 1‐alpha protein abundance. These log‐transformed data are presented as the geometric mean ± 95% confidence interval of the geometric mean. If the log‐transformed data were not normally distributed, then the original (raw) data were analysed using the non‐parametric Friedman's test and Wilcoxon signed rank tests to identify any changes over time or differences between trials. These data included the following: plasma glutathione peroxidase activity; TP53, thioredoxin and NAD(P)H quinone dehydrogenase 1 mRNA expression; and phosphorylated adenosine monophosphate‐activated protein kinase (p‐AMPK) protein abundance and the ratio of p‐AMPK:total AMPK. These data are presented as the median ± interquartile range. Adjustments for multiple comparisons were made using the false discovery rate (Curran‐Everett, 2000). Associations between fold‐changes in mitochondrial enzyme/complex abundance or activity and muscle oxygenation/oxidative capacity were examined using Pearson's correlation. Statistical significance was set at *P* < 0.05. Cohen's *d* was interpreted as small (0.2 ≤ *d* < 0.5), medium (0.5 ≤ *d* < 0.8) or large (*d* ≥ 0.8).

## 3 RESULTS

### 3.1 Blood oxidative stress markers after acute resistance exercise

**FIGURE 2:**
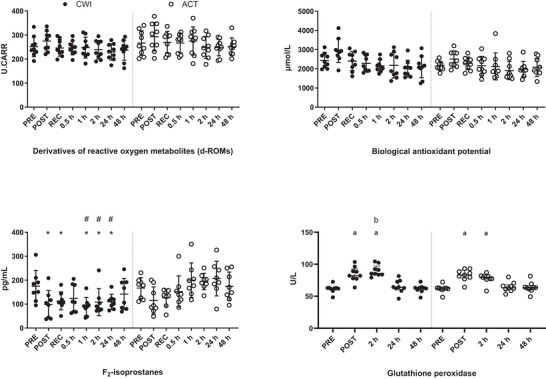
Blood oxidative stress biomarkers. *n* = 9 for CWI and ACT trials. Data for serum derivatives of reactive oxygen metabolite concentration, plasma F_2_‐isoprostane concentration and serum biological antioxidant potential are presented as the mean ± SD. Data for plasma glutathione peroxidase activity are presented as the median ± interquartile range. ^*^ *P* ≤ 0.007 versus pre‐exercise. ^#^ *P* ≤ 0.007 versus ACT. ^a^ *P* ≤ 0.0125 versus pre‐exercise. ^b^ *P* ≤ 0.0125 versus ACT. Abbreviations: ACT, active recovery; CWI, cold water immersion; REC, time point immediately after recovery treatments.

Data for blood oxidative stress markers are shown in Figure 2. Serum d‐ROMs concentration (time effect *P* = 0.002) and serum biological antioxidant potential (time effect *P* < 0.001) changed over time. However, no pairwise differences remained significant after false discovery rate correction, and these responses were not different between trials (time × trial effect *P* = 0.96 and *P* = 0.84, respectively). Plasma F_2_‐isoprostanes concentration differed over time (time effect *P* = 0.012), and this response differed between trials (time × trial effect *P* = 0.019). Values were lower 1 h (*P* = 0.006; *d* = −1.38), 2 h (*P* = 0.005; *d* = −1.40) and 24 h (*P* = 0.002; *d* = −1.77) after exercise in the CWI trial compared with the ACT trial. Plasma glutathione peroxidase activity changed over time (Friedman's test *P* < 0.001). Values were higher 2 h after exercise in the CWI trial compared with the ACT trial (*P* = 0.008; *d* = 1.36).

### 3.2 Redox‐regulated genes in skeletal muscle after acute resistance exercise

**FIGURE 3:**
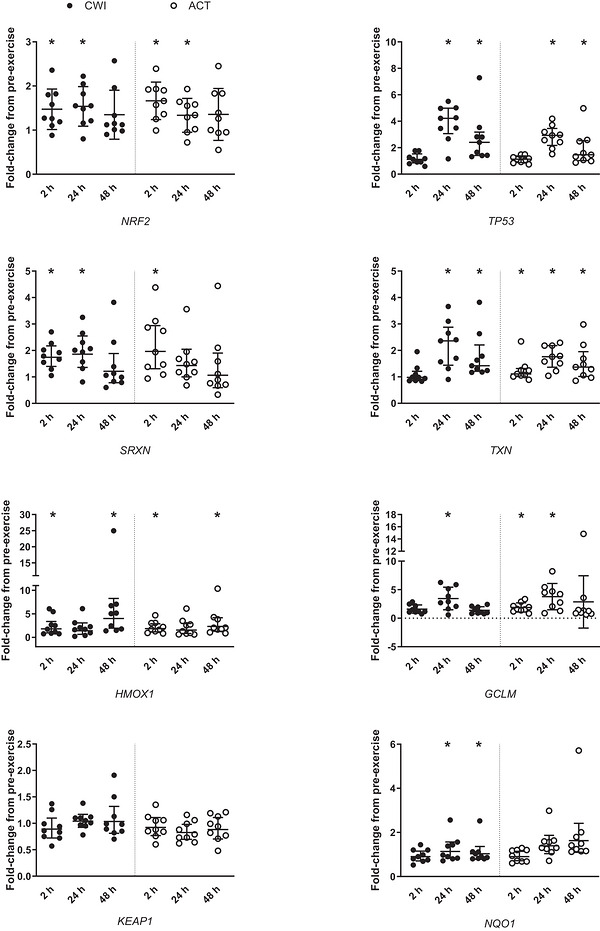
Expression of redox‐regulated genes in skeletal muscle. n = 9 for CWI and ACT trials. Data for *NRF2*, *GCLM* and *KEAP1* are presented as the mean ± SD. Data for *SRXN* and *HMOX1* are presented as the geometric mean ± 95% confidence interval of the geometric mean. Data for *TP53*, *NQO1* and *TXN* are presented as the median ± interquartile range. ^*^ *P* ≤ 0.033 versus pre‐exercise. Abbreviations: ACT, active recovery; CWI, cold water immersion; *GCLM*, glutamate–cysteine ligase modifier subunit; *HMOX1*, heme oxygenase 1; *KEAP1*, Kelch‐like ECH‐associated protein 1; *NQO1*, NAD(P)H quinone dehydrogenase 1; *NRF2*, nuclear factor erythroid 2‐related factor 2; *SRXN*, sulfiredoxin; *TP53*; *TXN*, thioredoxin.

Data for selected redox‐regulated genes are shown in Figure 3. The mRNA expression of nuclear factor erythroid 2‐related factor 2 (*NRF2*; time effect *P* < 0.001), sulfiredoxin 1 (*SXRN*; time effect *P* < 0.001), heme oxygenase 1 (*HMOX1*; time effect *P* < 0.001) and glutamate–cysteine ligase modifier subunit (*GCLM*; time effect *P* = 0.034) differed over time. However, these responses were not different between the two trials (time × trial effects *P* > 0.05). The mRNA expression of *TP53* (Friedman's test *P* < 0.001), thioredoxin (*TXN*; Friedman's test *P* < 0.001) and NAD(P)H quinone dehydrogenase 1 (*NQO1*; Friedman's test *P* = 0.004) also changed over time. The mRNA expression of Kelch‐like ECH‐associated protein 1 (*KEAP1*) did not change over time (time effect *P* = 0.50).

Expression of other genes, including glutathione reductase (*GSR*; time effect *P* = 0.021), thioredoxin reductase (*TXNRD*; time effect *P* = 0.010), superoxide dismutase 1 (*SOD1*; time effect *P* = 0.015) and glutathione peroxidase (*GPX*; time effect *P* = 0.039), also differed over time. However, no pairwise differences remained significant after false discovery rate correction (*P* ≤ 0.033), and these data are not shown. The mRNA expression of glutathione *S*‐transferase alpha (*GSTA*; time effect *P* = 0.50) and superoxide dismutase 2 (*SOD2*; time effect *P* = 0.85) did not change over time (data not shown).

### 3.3 Activity and protein abundance of mitochondrial enzyme complexes and citrate synthase in skeletal muscle after resistance training

**FIGURE 4:**
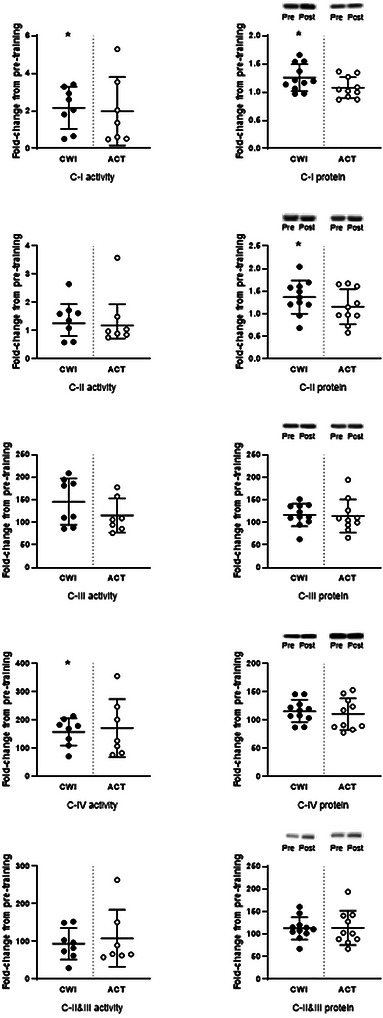
Fold‐changes in the abundance and activity of mitochondrial enzyme complexes. Protein abundance was normalised to the housekeeping protein GAPDH. For citrate synthase, C‐I and C‐II protein abundance, *n* = 10 for the ACT group and *n* = 11 for the CWI group. For citrate synthase, C‐I and C‐II enzyme activity, *n* = 7 for the ACT group and *n* = 8 for the CWI group. All data other than C‐II activity are presented as the mean ± SD. Data for C‐II activity are presented as the geometric mean ± 95% confidence interval of the geometric mean. ^*^ *P* ≤ 0.025 versus pretraining. Abbreviations: ACT, active recovery; CWI, cold water immersion.

Data for the activity and protein abundance of mitochondrial enzyme complexes are presented in Figure 4. The activity of C‐I (time effect *P* = 0.005) and C‐IV (time effect *P* = 0.023) changed over time; however, these responses were not different between the two groups (time × group effects *P* = 0.68 for C‐I and *P* = 0.83 for C‐IV). The activity of C‐II (time effect *P* = 0.12), C‐III (time effect *P* = 0.13) and C‐II+III (time effect *P* = 0.87) did not change over time.

The protein abundance of C‐I (time effect *P* = 0.002) and C‐II (time effect *P* = 0.001) differed over time; however, these responses were not different between the two groups (time × group effects *P* = 0.14 for C‐I and *P* = 0.31 for C‐II). Changes in the protein abundance of C‐III (time effect *P* = 0.051), C‐IV (time effect *P* = 0.051) and C‐II+III (time effect *P* = 0.098) did not reach statistical significance.

**FIGURE 5:**
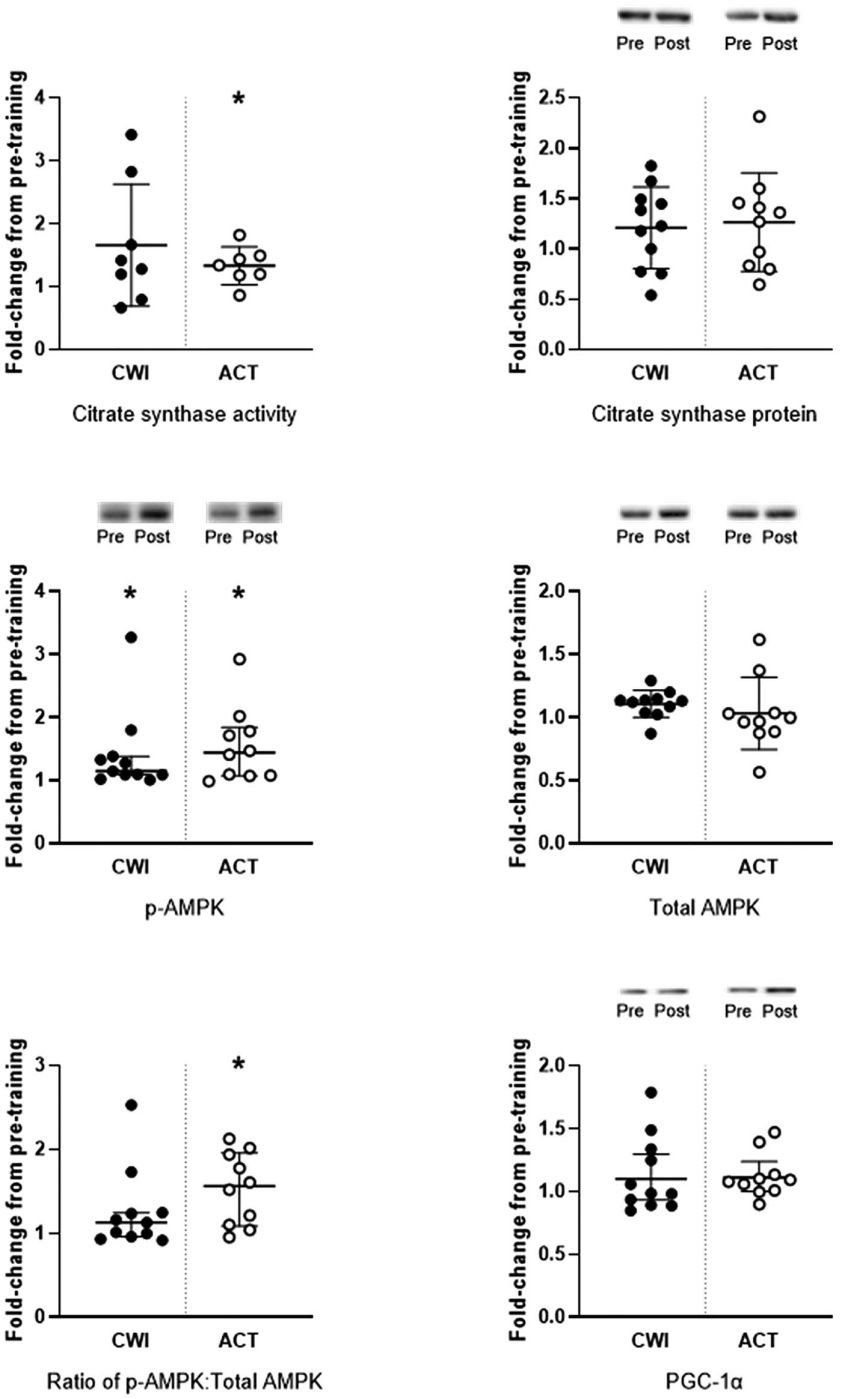
Fold‐changes in the activity and abundance of citrate synthase and regulators of mitochondrial biogenesis in skeletal muscle. Protein abundance was normalised to the housekeeping protein GAPDH. n = 10 for the ACT group and *n* = 11 for the CWI group. Data for citrate synthase protein, citrate synthase activity and total AMPK protein are presented as the mean ± SD. Data for PGC‐1α protein are presented as the geometric mean ± 95% confidence interval of the geometric mean. Data for p‐AMPK protein and the ratio of p‐AMPK:total AMPK are presented as the median ± interquartile range. ^*^ *P* ≤ 0.025 versus pretraining. Abbreviations: ACT, active recovery; CWI, cold water immersion; AMPK, adenosine monophosphate‐activated protein kinase; PGC‐1α, peroxisome proliferator‐activated receptor gamma coactivator 1‐alpha.

Citrate synthase activity differed over time (time effect *P* = 0.044); however, this response was not different between the two groups (time × group effect *P* = 0.81) (Figure 5). Changes in the protein abundance of citrate synthase did not reach statistical significance (time effect *P* = 0.76).

### 3.4 Protein abundance of AMPK, PGC‐1α and PPAR‐α after resistance training

Data for protein abundance of AMPK and PGC‐1α protein are shown in Figure 5. The p‐AMPK protein abundance changed over time (Friedman's test *P* < 0.001), whereas total AMPK protein abundance did not change (time effect *P* = 0.12). The p‐AMPK:total AMPK ratio also differed over time (Friedman's test *P* = 0.019). Changes in protein abundance of PGC‐1α did not reach statistical significance (time effect *P* = 0.067).

### 3.5 Muscle oxygenation and oxidative capacity after resistance training

**FIGURE 6:**
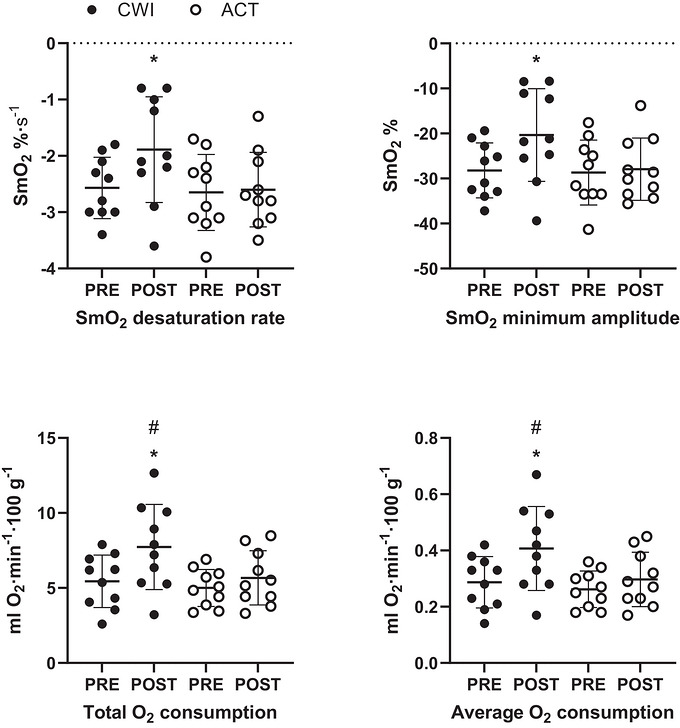
Muscle oxygenation and oxidative capacity. *n* = 10 for CWI and ACT groups. Data are presented as the mean ± SD. ^*^ *P* ≤ 0.025 versus pretraining. Abbreviations: CWI, cold water immersion; ACT, active recovery; $S_{mO_{2}}$, muscle oxygen saturation.

Data for muscle oxygenation and oxidative capacity are shown in Figure 6. Muscle oxygen desaturation rate (time effect *P* = 0.046) and the minimum amplitude for muscle oxygenation (time effect *P* = 0.024) during the 10‐s isometric muscle contraction changed over time; however, these responses did not differ between the two groups (time × group effect *P* = 0.070 for desaturation rate and *P* = 0.054 for the minimum amplitude).

Total muscle oxygen consumption during the isokinetic contractions changed over time (time effect *P* = 0.008), and this response differed between the two groups (time × group effect *P* = 0.001). It increased in the CWI group (*P* = 0.001; *d* = 1.43) but not in the ACT group (*P* = 0.15) after training. It was higher in the CWI group compared with the ACT group after training (*P* = 0.023; *d* = 0.86). Average muscle oxygen consumption during the isokinetic contractions also changed over time (time effect *P* = 0.010), and this response differed between the two groups (time × group effect *P* = 0.002). It increased in the CWI group (*P* = 0.002; *d* = 1.36), but not the ACT group (*P* = 0.16) after training. It was higher in the CWI group compared with the ACT group after training (*P* = 0.021; *d* = 0.88).

**FIGURE 7:**
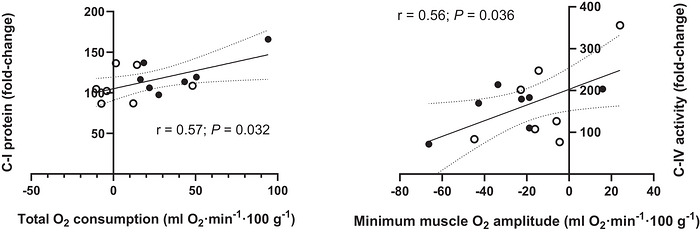
Scatter plots showing relationships between pre–post training fold changes in complex I (C‐I) protein abundance and post‐training total muscle O_2_ consumption, and between pre–post training fold changes in complex IV (C‐IV) activity and post‐training minimum O_2_ amplitude. Pearson correlation coefficients (*r*) are shown in each panel. Dotted lines indicate the 95% confidence intervals. Open circles represent the active recovery group. Filled circles represent the cold water immersion group.

Associations between fold‐changes in mitochondrial enzyme/complex protein abundance or activity and measures of muscle oxygenation or oxidative capacity are shown in Table 2. Across the participants, the training‐induced change in C‐I protein abundance was positively correlated with total muscle oxygen consumption during the isokinetic contractions (*r* = 0.574, *P* = 0.032; Figure 7). Likewise, the training‐induced change in C‐IV activity was positively correlated with the minimum muscle oxygenation amplitude during the isometric contractions (*r* = 0.563, *P* = 0.036; Figure 7).

**TABLE 2:** Correlation matrix for relationships between changes in mitochondrial enzymes, regulators of mitochondrial biogenesis, muscle oxidative capacity and oxygen consumption after training.

	Citrate synthase activity	Citrate synthase abundance	C‐I activity	C‐I abundance	C‐IV activity	O_2_ desaturation	O_2_ minimum amplitude	Total O_2_ consumption	p‐AMPK abundance	PGC‐1α abundance
Citrate synthase activity	1.00	*r* = −0.061 *P* = 0.84	*r* = −0.37 *P* = 0.19	*r* = −0.12 *P* = 0.69	*r* = −0.36 *P* = 0.21	*r* = −0.52 *P* = 0.06	*r* = −0.50 *P* = 0.07	*r* = −0.29 *P* = 0.31	*r* = −0.32 *P* = 0.26	*r* = 0.33 *P* = 0.25
Citrate synthase abundance	–	1.00	*r* = −0.27 *P* = 0.36	*r* = 0.61 *P* = 0.02	*r* = −0.082 *P* = 0.78	*r* = −0.12 *P* = 0.68	*r* = −0.18 *P* = 0.54	*r* = 0.21 *P* = 0.46	*r* = 0.36 *P* = 0.21	*r* = 0.34 *P* = 0.24
C‐I activity	–	–	1.00	*r* = −0.33 *P* = 0.25	*r* = −0.18 *P* = 0.53	*r* = −0.11 *P* = 0.72	*r* = −0.10 *P* = 0.75	*r* = 0.14 *P* = 0.64	*r* = −0.004 *P* = 0.99	*r* = −0.45 *P* = 0.11
C‐I abundance	–	–	–	1.00	*r* = 0.008 *P* = 0.98	*r* = −0.15 *P* = 0.60	*r* = −0.15 *P* = 0.61	*r* = 0.57 *P* = 0.032	*r* = 0.12 *P* = 0.69	*r* = 0.15 *P* = 0.60
C‐IV activity	–	–	–	–	1.00	*r* = 0.53 *P* = 0.052	*r* = 0.56 *P* = 0.036	*r* = −0.23 *P* = 0.43	*r* = −0.011 *P* = 0.97	*r* = −0.36 *P* = 0.21
O_2_ desaturation	–	–	–	–	–	1.00	*r* = 0.99 *P* < 0.001	*r* = −0.18 *P* = 0.54	*r* = 0.084 *P* = 0.78	*r* = −0.17 *P* = 0.55
O_2_ minimum amplitude	–	–	–	–	–	–	1.00	*r* = −0.20 *P* = 0.49	*r* = 0.052 *P* = 0.86	*r* = −0.25 *P* = 0.38
Total O_2_ consumption	–	–	–	–	–	–	–	1.00	*r* = 0.081 *P* = 0.78	*r* = −0.13 *P* = 0.67
p‐AMPK abundance	–	–	–	–	–	–	–	–	1.00	*r* = 0.34 *P* = 0.23
PGC‐1α abundance	–	–	–	–	–	–	–	–	–	1.00

*Note*: Only variables that demonstrated a significant change after training were included in this analysis.

## 4 DISCUSSION

There were two aims in this study. First, we compared how CWI and ACT following a single resistance exercise session influence short‐term changes in blood oxidative stress markers and redox‐regulated gene expression in skeletal muscle. Second, we compared how regular, repeated CWI and ACT affect long‐term adaptations in mitochondrial enzymes, regulators of mitochondrial biogenesis and oxidative capacity in skeletal muscle after 3 months of resistance training. Addressing these aims together allowed us to evaluate whether early redox‐related signals aligned with subsequent mitochondrial and muscle oxygenation adaptations. Contrary to our hypotheses, there were no substantial or consistent differences between the CWI and ACT treatments. These findings suggest that physiological responses to CWI (e.g., reduced venous blood O_2_ saturation, reduced muscle blood flow and changes in muscle perfusion) do not modify systemic oxidative stress, redox‐related gene expression or mitochondrial adaptations in skeletal muscle after acute and chronic resistance exercise. We did observe that regular CWI enhanced training‐induced oxidative metabolic capacity in muscle, which might have implications for resistance to fatigue during exercise.

Exercise‐induced oxidative stress is commonly evaluated by measuring markers of lipid, protein and DNA damage, in addition to the concentration of antioxidants and activity of antioxidant enzymes in blood or urine. We observed a moderate (non‐significant) increase in plasma d‐ROMs concentration immediately after exercise, reflecting the presence of hydroperoxides. Conversely, the concentration of plasma F_2_‐isoprostanes, a marker of lipid peroxidation, decreased unexpectedly after exercise. These findings differ from earlier studies reporting increased concentrations of F_2_‐isoprostanes in urine (Rietjens et al., 2007), and plasma malondialdehyde (Goldfarb et al., 2005; Mohammadjafari et al., 2019) and thiobarbituric acid reactive substances (Deminice et al., 2010) after resistance exercise. This variability in results is likely to stem, in part, from differences in the chemistries underlying these oxidative stress biomarkers. We measured plasma F_2_‐isoprostanes concentration at seven time points from immediately after exercise up to 48 h. Although plasma F_2_‐isoprostanes concentration might have peaked between these sampling time points, the relatively high sampling frequency across the early and late recovery period makes it unlikely that we missed a substantial response. We found large increases in serum biological antioxidant potential and glutathione peroxidase activity following exercise. These responses align with previous research showing greater total antioxidant capacity (Hudson et al., 2008; Rietjens et al., 2007), reduced glutathione (Deminice et al., 2010), oxidised glutathione (Goldfarb et al., 2005), oxidised:total gluthathione (Goldfarb et al., 2008), glutathione peroxidase and catalase activity (Mohammadjafari et al., 2019) in plasma after resistance exercise.

Few studies have investigated how CWI affects systemic oxidative stress after exercise (Coelho et al., 2021; de Freitas et al., 2019; Leeder et al., 2019). In our study, plasma F_2_‐isoprostanes concentration was lower, whereas plasma glutathione peroxidase activity was higher following CWI compared with ACT. The lower plasma concentration of F_2_‐isoprostanes in the CWI trial might suggest reduced oxidative stress. However, changes in F_2_‐isoprostanes might also reflect alterations in antioxidant activity (e.g., glutathione peroxidase) or lipid peroxidation dynamics, rather than a simple reduction in oxidative stress per se. Others have found no difference in the concentration of plasma lipid hydroperoxides after postexercise CWI compared with passive recovery (Leeder et al., 2019). These discrepancies underscore the need for further research to clarify how CWI influences biomarkers of exercise‐induced oxidative stress.

To complement the systemic oxidative stress measurements, we examined redox‐related transcriptional responses within skeletal muscle. We present new evidence that the expression of *NRF2* and several of its downstream targets, including *HMOX1*, *NQO1* and *GCLM*, increased in skeletal muscle after resistance exercise. NRF2 is a transcription factor that is integral in regulating the antioxidant response to various cellular stressors, including reactive oxygen species (Powers et al., 2024). In turn, these antioxidant effects of NRF2 protect muscle cells from cytotoxicity (Horie et al., 2015; Pribil Pardun et al., 2024). NRF2 expression and binding to the antioxidant response element in muscle cells depends on downregulation of KEAP1 (Ostrom et al., 2021). Of note, we found that *KEAP1* expression in skeletal muscle did not change after exercise (time effect *P* = 0.57), which might have permitted *NRF2* expression. One other study has reported that *HMOX1* expression increased in skeletal muscle after eccentric exercise (MacNeil et al., 2010). HMOX1 is a downstream target of NRF2, but it is also influenced by several other transcription factors (Xiao et al., 2023). It defends muscle cells from cytotoxicity and DNA damage (Choi, 2020; Wilson et al., 2015). It also protects muscle from ischaemia–reperfusion injury (Wilson et al., 2015). Other research has shown that *NQO1* is expressed in contracting muscle cells (Horie et al., 2015; Ostrom et al., 2021), mouse skeletal muscle (Yamada et al., 2019) and human peripheral blood mononuclear cells (Komine et al., 2021) after exercise. By maintaining the reduced forms of antioxidant molecules, such as ubiquinone (coenzyme Q) and vitamin E, NQO1 contributes to cellular antioxidant capacity, which safeguards cells from oxidative damage. NQO1 influences the cellular NAD^+^:NADH ratio, which is vital for various metabolic processes and the maintenance of redox homeostasis (Ross & Siegel, 2017). *TP53* mRNA expression increased in muscle after both trials. Others have reported increased expression of p53 protein expression in muscle after resistance exercise (Camera et al., 2015). At low levels of oxidative stress, p53 activates pathways that increase time for cell repair (e.g., cell cycle arrest and autophagy) to enhance cell survival. At higher levels of oxidative stress, p53 facilitates increased cellular stress by initiating DNA fragmentation to induce apoptosis. In turn, this prevents aberrant cell proliferation (Beyfuss & Hood, 2018).

In addition to *NRF2*, *NQO1*, *HMOX1* and *TP53*, we discovered that the expression of several other redox‐regulated genes, including *GCLM*, *TXN* and *SRXN1*, increased in skeletal muscle after resistance exercise. Others have demonstrated that *Gclm* expression increased after exercise in skeletal muscle of mice (Li et al., 2015). GCLM is a crucial component of the enzyme glutamate–cysteine ligase, which is the rate‐limiting enzyme in glutathione biosynthesis (Yang et al., 2002). One other study reported no change in *TXN* expression in skeletal muscle after endurance exercise (Chaves et al., 2022). TXN plays a crucial role in maintaining protein thiol homeostasis and regulating reactive oxygen species signalling in both intracellular and extracellular environments (Tinkov et al., 2018). It also influences the activity of several transcription factors, including nuclear factor‐κB (Hirota et al., 1999). *SRXN1* has not been reported in the skeletal muscle or exercise literature. It attenuates oxidative stress and inflammation in cardiac muscle by increasing *SIRT1* expression and reducing *NLRP3* inflammasome activation (Zhang et al., 2024). There were no significant differences in these redox‐related transcriptional responses between the CWI and ACT trials.

As a marker of mitochondrial volume/content and activity (Larsen et al., 2012), the activity of citrate synthase and electron transport chain complexes C‐I and C‐IV increased in skeletal muscle after resistance training. However, these responses did not differ between the ACT and CWI groups. These findings suggest that regular CWI did not meaningfully alter adaptations in muscle mitochondrial volume/content. Previous literature on changes in citrate synthase activity in skeletal muscle after resistance training in young adults is inconsistent, with reports of an increase (Tang et al., 2006), a decrease (Haun et al., 2019; Kon et al., 2014; Roberts et al., 2018) or no change (Groennebaek et al., 2018; Porter et al., 2015; Ruple et al., 2021). Porter et al. (2015) also reported increased C‐I protein abundance, but no change in C‐II, C‐III, C‐IV or C‐V protein abundance in muscle after 3 months of resistance training. Using high‐resolution respirometry, Groennebaek et al. (2018) showed that 6 weeks of resistance training increased mitochondrial respiratory function in permeabilised muscle fibres. The inconsistencies reported in the literature about the effects of resistance training on mitochondrial content/activity are likely to reflect variation in training regimens and methods to quantify mitochondrial volume/content and activity in skeletal muscle (Parry et al., 2020). The mitochondrial responses that we discovered align with the minimal differences observed in acute redox‐regulated gene expression, suggesting that redox‐dependent signalling did not drive treatment‐specific adaptations.

We found that the protein abundance of p‐AMPK increased in skeletal muscle after resistance training. Other studies on chronic resistance training have reported no change in p‐AMPK protein abundance in skeletal muscle (Li et al., 2012; Vissing et al., 2013). The disparity between our findings and these other studies might be attributable to differences in the age and training status of the participants. Our data indicated a similar increase in p‐AMPK in the CWI and ACT groups after training. Previous research investigating the effects of CWI on p‐AMPK after aerobic exercise has reported inconsistent results (Aguiar et al., 2016; Ihsan et al., 2015).

Relatively little research has examined how chronic resistance training influences muscle oxygenation and oxidative capacity measured with NIRS. One study reported that muscle deoxygenation during 7 min of occlusion of the vastus lateralis increased by 39% after 6 weeks of strength training (three times per week) (Uchiyama et al., 2011). Another study reported that the decline in muscle oxygen saturation during a 30‐s isometric contraction (at 20% maximum voluntary contraction) was greater in trained versus untrained individuals (Lin et al., 2014). We present new evidence that muscle oxygen desaturation and the minimum amplitude for muscle oxygenation during a 10‐s isometric muscle contraction decreased after training in the CWI group. We also discovered, for the first time, that the average and total muscle oxygen consumption during 50 maximal isokinetic contractions increased after training in the CWI group. The slower oxygen desaturation rate and higher minimum muscle oxygenation in the CWI group after training suggest improvements in oxygen delivery and utilisation during resistance exercise. When considered alongside the increase in average and total muscle oxygen consumption in this group, these data are likely to reflect an overall enhancement in oxidative metabolic capacity.

The moderate correlations we discovered between training‐induced changes in C‐I protein abundance and total muscle oxygen consumption, and between C‐IV activity and the minimum muscle oxygenation amplitude during contractions (Figure 7), suggest that individuals with greater enhancement of electron transport chain activity also exhibited larger improvements in muscle oxygen handling. These correlation analyses were exploratory, and *P*‐values were not adjusted for multiple comparisons; therefore, these findings should be interpreted with caution.

The improvements in oxygen delivery and utilisation that we observed are likely to reflect broader features of muscle structure and phenotype. Others have proposed that a greater proportion of type II fibres in contracting muscle might reduce the microvascular O_2_ pressure and thereby increase O_2_ extraction (Lin et al., 2014). Consistent with this notion, we previously reported that after training in these same groups of participants, the proportion of type IIa myofibres increased in the CWI and ACT groups (D'Souza et al., 2018). Concomitantly, the expression of *MYH2* in muscle (which encodes type IIa fibres) decreased in the ACT group, whereas it remained unchanged in the CWI group after training (D'Souza et al., 2018). Regular CWI might therefore have helped to preserve the transcriptional profile of type IIA fibres during fibre‐type transitions, potentially contributing to the greater improvements in muscle metabolic capacity that we observed in this group. We also previously documented that the number of capillaries per muscle fibre increased after training in the CWI group (D'Souza et al., 2018). This might have enhanced oxygen delivery to muscle fibres, which could also account for our present observation that muscle metabolic capacity improved after training in the CWI group. Enhanced oxygenation dynamics indicate better coupling of oxygen delivery and utilisation during contractions, which could, in turn, support more stable intramuscular energetics and (potentially) delay the onset of fatigue during repeated or sustained efforts.

A key strength of this study is the combination of acute and long‐term analyses, which allowed us to assess both acute molecular responses and chronic physiological adaptations to resistance training. The use of multiple measurement techniques, including gene expression, enzyme activity assays and NIRS, provided a comprehensive evaluation of muscle redox status and metabolic capacity. However, the study is not without limitations. The blood‐based markers that we measured might not fully capture local oxidative stress responses within muscle. Additionally, the enzymatic assays that we used to measure mitochondrial content and activity might not reflect functional changes detectable by more advanced methods, such as high‐resolution respirometry. We recruited only male participants, which limits the generalisability of our findings to females. Finally, although the study was adequately powered for primary outcomes, some findings should be interpreted cautiously owing to variability and small effect sizes.

## 5 CONCLUSION

In summary, in this study we investigated the acute and long‐term effects of resistance exercise combined with CWI compared with ACT on redox‐related transcriptional responses and metabolic adaptations to resistance training. Despite clear exercise‐induced changes in systemic oxidative stress markers and redox‐regulated gene expression, there were no substantial or consistent differences between the recovery treatments. We observed changes in muscle oxygenation and oxygen consumption consistent with improved oxidative capacity following resistance training, particularly in the CWI group. Collectively, our findings indicate that CWI does not impair redox‐related transcriptional responses or mitochondrial adaptations to resistance exercise and, in some contexts, might support enhanced oxygen handling and metabolic function in skeletal muscle. Importantly, combining both acute and chronic measures was essential for identifying this pattern, revealing that similar redox signatures can accompany distinct longer‐term metabolic outcomes in skeletal muscle.

## AUTHOR CONTRIBUTIONS

Llion A. Roberts, Truls Raastad, Jeff S. Coombes, David Cameron‐Smith and Jonathan M. Peake conceived and designed the work. Llion A. Roberts, Chantal A. Pileggi, Nina Zeng, Vandre C. Figueiredo, James F. Markworth, Truls Raastad, David Briskey, Katsuhiko Suzuki and Jonathan M. Peake acquired, analysed and/or interpreted data for the work. Llion A. Roberts, Vandre C. Figueiredo, James F. Markworth, David Cameron‐Smith and Jonathan M. Peake drafted and critically revised the work. All authors have approved the final version of the manuscript and agree to be accountable for all aspects of the work in ensuring that questions related to the accuracy or integrity of any part of the work are appropriately investigated and resolved. All persons designated as authors qualify for authorship, and all those who qualify for authorship are listed.

## CONFLICT OF INTEREST

None declared.

## GENERATIVE AI STATEMENT

The authors acknowledge their use of ChatGPT5.2 (accessed October−December 2025) to generate the Highlights summary and for feedback on the cohesion and organisation of the manuscript.

## Data Availability

The authors declare that all the original data related to the results presented in this study are available upon request.
